# Chalcone Synthase (CHS) Gene Suppression in Flax Leads to Changes in Wall Synthesis and Sensing Genes, Cell Wall Chemistry and Stem Morphology Parameters

**DOI:** 10.3389/fpls.2016.00894

**Published:** 2016-06-24

**Authors:** Magdalena Zuk, Magdalena Działo, Dorota Richter, Lucyna Dymińska, Jan Matuła, Andrzej Kotecki, Jerzy Hanuza, Jan Szopa

**Affiliations:** ^1^Department of Genetic Biochemistry of Plants, Faculty of Biotechnology, Wroclaw University, WroclawPoland; ^2^Linum Foundation, WroclawPoland; ^3^Department of Botany and Plant Ecology, Wroclaw University of Environmental and Life Sciences, WroclawPoland; ^4^Department of Bioorganic Chemistry, Institute of Chemistry and Food Technology, Faculty of Economics and Engineering, University of Economics, WroclawPoland; ^5^Institute of Biology, Wroclaw University of Environmental and Life Sciences, WroclawPoland; ^6^Department of Crop Production, Wroclaw University of Environmental and Life Sciences, WroclawPoland; ^7^Department of Genetics, Plant Breeding and Seed Production, Wroclaw University of Environmental and Life Sciences, WroclawPoland

**Keywords:** chalcone synthase, lignin biosynthesis, cell wall, flax, flavonoid

## Abstract

The chalcone synthase (CHS) gene controls the first step in the flavonoid biosynthesis. In flax, CHS down-regulation resulted in tannin accumulation and reduction in lignin synthesis, but plant growth was not affected. This suggests that lignin content and thus cell wall characteristics might be modulated through CHS activity. This study investigated the possibility that CHS affects cell wall sensing as well as polymer content and arrangement. CHS-suppressed and thus lignin-reduced plants showed significant changes in expression of genes involved in both synthesis of components and cell wall sensing. This was accompanied by increased levels of cellulose and hemicellulose. CHS-reduced flax also showed significant changes in morphology and arrangement of the cell wall. The stem tissue layers were enlarged averagely twofold compared to the control, and the number of fiber cells more than doubled. The stem morphology changes were accompanied by reduction of the crystallinity index of the cell wall. CHS silencing induces a signal transduction cascade that leads to modification of plant metabolism in a wide range and thus cell wall structure.

## Introduction

Plants produce more than 10 000 phenolics with diverse structures and properties (Kumar Anal et al., 2014). The main classes of flax phenolics are: the simple phenolics, such as phenolic acids and benzoic acid derivatives; flavonoids such as vitexin; tannins such as catechin derivatives; and lignin precursors such as coniferyl aldehyde. All these compounds share the basic phenolic structure, i.e., a six-carbon phenyl ring to which a three-carbon side chain is attached in a group of phenylpropanoids (Li et al., 2010). Plant phenolics are synthesized from precursors, and the starting point is the shikimic acid pathway. Two streams of compound synthesis have been suggested. The main stream is the formation of chorismic acid, which is subsequently metabolized to aromatic amino acids; this is an essential route of phenylpropanoid synthesis. The remaining stream leads to gallic acid synthesis and hydrolysable tannin formation. The first step in the phenylpropanoid pathway is catalyzed by phenylalanine ammonia lyase (PAL), which converts phenylalanine to trans-cinnamic acid. C4H forms p-coumaric acid, which is then converted to p-coumaryl-CoA by 4-coumarate CoA ligase (4CL). The enzyme cinnamate-3-hydroxylase (C3H) introduces a hydroxyl group into p-coumaric acid and upon methylation converts it into ferulic acid. Condensation of p-coumaryl-CoA and malonyl-CoA catalyzed by CHS is the first step common to all flavonoid biosynthesis. The route is completed with synthesis of several compounds including condensed tannins (proanthocyanidins). The essential step in their synthesis is the condensation of flavan-3-ols such as catechin and epicatechin.

Monolignols such as p-coumaryl, coniferyl and sinapyl alcohols are the substrate for lignin biosynthesis (Shinya et al., 2014). These monomers are formed from coumaric acid precursors by hydroxylation and *O*-methylation reactions catalyzed by *O*-methyltransferase. Then these intermediates are converted to aldehyde and alcohol forms. Finally the lignin formation is the result of chemical coupling of monolignol radicals. The reaction is catalyzed by peroxidase and H_2_O_2_ or laccases.

Linseed flax is predominantly a source of valuable oil, highly enriched with omega-3 fatty acids for review see Zuk et al. (2015). Besides oil, the linseed plant provides fibers, seedcake, and shives as byproducts. The recent development of analytical methods allows identification of several valuable compounds of different pathways. Besides lignocellulosic biomass mainly used for components of polymer composites and liquid/gas fuel production, phenylpropanoids and terpenoids are the constituents that provide biological properties to the byproducts. Antibacterial, antifungal, anticancer, and anti-inflammatory activities are assigned to these compounds (Czemplik et al., 2011; Zuk et al., 2014).

Genetic engineering, to alter a single or multiple reaction steps in these pathways, provides a reasonable experimental approach to understand the regulation of these pathways and their cross-talk with pathways whose products occupy the same or a similar cell compartment. These approaches might result in the production of new plants and products. Indeed, recently generated linseed plants with a down-regulated CHS gene (W86 plant) resulted in tannin accumulation in seed and changes in the ω6 to ω3 fatty acid ratio in oil, beneficial for human diet (Zuk et al., 2012). Thus CHS suppression is beneficial for industries and diversifies linseed plant application. It was interesting that overproduction of tannin resulted in lignin reduction. Since lignin is a constituent of the cell wall, it was of interest whether it affects the level and biosynthesis of other cell wall constituents. Thus in this study we investigated expression of genes involved in cell wall polymer metabolism, cell wall constituent levels, cell wall polymer arrangement and morphology of the cell wall in 5th generation W86 linseed plants cultivated in a field at the end of the vegetation period (13th week of growth, almost-mature stage of growth). The data obtained strongly suggest that the CHS gene is involved in cell wall polymer biosynthesis and arrangement.

## Materials and Methods

### Plant Material

Chalcone synthase gene down-regulation was performed according to a procedure described previously (Zuk et al., 2012). Fifth-generation transgenic flax (line W86) and control plants were field cultivated. The flax plants were harvested at the end of the vegetation period (August 2015) in the almost-mature stage of growth (13th week of growth). At this stage plants are fully developed, seed capsules are shaped but plants are still green. The stalks (the middle part of the stem, without seed capsules, flowers, or roots) were collected. The metabolism was quenched by shock-freezing of plant tissue in liquid nitrogen, followed by grinding of the frozen material. The collected material was divided into two samples – one for gene expression analysis and the other for metabolite analysis (this sample was freeze-dried).

### Cellulose Content

The cellulose content was determined using the colorimetric method with the reagent anthrone, as described by Ververis et al. (2004). 15 mg of dry, ground flax stalk were incubated with a mixture of nitric and acetic acid (1:8 v/v) for 1 h at 100°C and then centrifuged (5 min, 14000 rpm). The pellet was washed twice with water and then resuspended in 1 ml of 67% H_2_SO_4_ (v/v). After mixing samples, cold anthrone reagent was added and the cellulose level in these samples was measured spectrophotometrically at 620 nm. Commercially available cellulose after hydrolysis was used for the calibration curve.

### Lignin Content

The analysis of the total lignin content was performed using the acetyl bromide method, according to the protocol described by Iiyama and Wallis (1990). 15 mg of dry, ground flax fibers were heated for 2 h at 100°C, then 10 ml water was added to each sample, and the samples were heated for 1 h at 65°C with mixing every 10 min. In the next step the samples were filtered through a GF/A glass fiber filter (Whatman) and rinsed triply with each of the following solutions: water, ethanol, acetone and diethyl ether. The filters were placed in glass vials and heated overnight at 70°C. Then 25% acetyl bromide (2.5 ml) in acetic acid was added and the vials were placed at 50°C for 2 h. The cooled samples were mixed with 10 ml of 2 M sodium hydroxide and 12 ml of acetic acid and were incubated at room temperature (RT) overnight. The lignin content was measured at 280 nm. Coniferyl alcohol was used to prepare a calibration curve.

### Pectin and Hemicellulose Content

In order to remove contamination 1 g of grounded stalk samples was extracted with 96% ethanol at 100°C, 80% ethanol at 80°C, chloroform: methanol solution (1:1 v/v) at 40°C and then acetone at RT. After drying, the samples were hydrolyzed with concentrated H_2_SO_4_ in an ice bath. The samples were diluted with water and centrifuged, and the supernatant containing pectin was collected in the new tubes. The remaining pellet was resuspended in 4 M KOH with 20 mM NaBH_4_, stirred at RT for 12 h and washed, and then supernatants were neutralized with HCl to yield the 4 M KOH-soluble fraction (hemicellulose).

The amount of pectin and hemicellulose was determined by the biphenyl method using a spectrophotometer. The hydrolysate was supported in turn with 4 M sulfamic acid potassium sulfonate solution, pH = 1.6, Na_2_B_4_O_7_ in H_2_SO_4_, then incubated for 20 min at 100°C. In the last step, *m*-hydroxybiphenyl was added to measure absorption at 525 nm. The results were given as an equivalent of glucuronic acid (Wojtasik et al., 2013).

### Phenolic Compound Extraction and Measurement by UPLC

0.5 g of lyophilized flax stalk was ground in a Retsch mill to a fine powder and then extracted three times with methanol using an ultrasonic bath (15 min). The samples were centrifuged (5 min, 5000 rpm, RT), and the supernatant was collected, evaporated under a vacuum and resuspended in 1 ml of methanol. The remaining pellet was hydrolyzed in 2 N NaOH at RT for 24 h to release bound phenolics. Extracts were adjusted to pH 3, and the samples were extracted three times with ethyl acetate and centrifuged (1 min, 5000 rpm, RT). The supernatants were collected, evaporated under a vacuum and resuspended in 1 ml of methanol. Then the samples were analyzed on a Waters Acquity UPLC system with a 2996 PDA detector, using an Acquity UPLC column BEH C18, 2.1 mm × 100 mm, 1.7 μm. The mobile phase was A = 0.1% formic acid and B = acetonitrile, in a gradient flow: 1 min at 95% A/5% B; 12 min gradient to 70% A/30% B; 15 min gradient to 0% A/100% B; and 17 min 95% A/5% B with a 0.1 ml/ min flow rate. Detection of the compounds: coumaric, caffeic, ferulic, and syringic acid and aldehyde, vanillin, vitexin, orientin, and isoorientin was done at 320 nm and that of vanillic acid at 280 nm.

### Identification of cDNA Sequences and Primer Design

Unknown cDNA sequences of the flax genes of interest (lacking an accession number) were identified based on homology alignments with the known gene sequences from other plant species. The primers were designed for the most homologous regions of the tested gene. The reaction product was analyzed via gel electrophoresis, and after extraction from the gel using a DNA Gel-out Kit it was cloned with a TOPO TA Cloning Kit (Invitrogen) and sequenced (Genomed SA). For verification the obtained DNA sequences were compared with the flax genome sequence (*Linum usitatissimum* cv. Bethune) and aligned with corresponding genes from other plants in the GenBank database^1^. Primers were designed on the most conservative regions of the genes or the regions presented in all known izoforms of examined enzyme and their sequences are presented in Supplementary Material (Preisner, Wojtasik Unpublished Data). Specific primers were designed using Primer-BLAST in NCBI. The criteria for primer design were set as follows: primer lengths of 20–24 bp, GC contents of 45–55%, melting temperature (Tm) in a range of 55–60°C and amplicon lengths of 100–250 bp.

### Gene Expression Analysis

The mRNA level for the investigated genes was determined using real-time PCR. For each analysis at least three independent biological samples were used (the middle part of stem. without seed capsules, flowers and roots). The 0.2 g of material was homogenized in liquid nitrogen to extract total RNA using the Trizol method (Invitrogen) following the manufacturer’s protocol. The remaining DNA was removed via DNase I (Invitrogen) treatment. Then, RNA was used as a template for cDNA synthesis using a High Capacity cDNA Reverse Transcription Kit (Applied Biosystems). The integrity of total RNA was verified by executing 1.5% (w/v) agarose gel electrophoresis, and the quantity and quality of RNA samples were tested with the NanoDrop 2000 Spectrophotometer (NanoDrop Technologies, ThermoScientific, USA). Only the RNA samples with absorption ratios of A260/280 = 1.8–2.2 and A260/230 higher than 1.8 were used for cDNA synthesis. The cDNA was diluted 20-fold with nuclease-free water for qRT-PCR. Real-time PCR reactions was conducted in 96-well plates using a DyNAmo SYBR Green qPCR Kit (Thermo Scientific) on the Applied Biosystems Step One Plus Real-Time PCR System. The reaction mix contained 2 μL diluted cDNA, 7.5 μL qPCR SYBR Green Master Mix (Thermo Scientific), 0.4 μM of each primer and ddH_2_O in a final volume of 15 μL. Reaction conditions were designed according to the kit manufacturer’s instructions. The qRT-PCR protocol was as follows: 95°C for 10 min, 40 cycles of 95°C for 15 s, 60°C for 30 s. To verify the specificity of each primer, a melting-curve analysis was included. Two biological replicates for all of the samples and three technical replicates of each biological replicate with a no-template control (NTC) were used. The actin gene was used as a reference gene. The changes in transcript levels were presented as the RQ to the reference gene.

### FT-IR and X-ray Analysis

Plant material (dried stalks from control and transgenic plants) was analyzed at RT in the spectral range 400–4000 cm^-1^ using an FT-IR Bio-Rad 575C spectrometer with a 2 cm^-1^ resolution. Approximately 0.25 g of each sample (control and transgenic flax stalks) were powdered in an automatic mortar grinder and pressed into a pellet using a hydraulic press. In the mid-IR region, the samples were prepared in KBr pellets.

Powder diffraction data were collected on an X’Pert PRO X-ray diffractometer with PIXcel ultra-fast line detector and Soller slits for Cu Kα radiation. The measurements were performed in transmission mode in the 5–80 2q range. Diffractograms were obtained on the same sample pellets that were analyzed in FT-IR. In order to calculate the crystallinity index (CI), the ratio of the crystallinity part of the 200 peak to the total absolute peak height was used (Keshk, 2015). The peak present at about 22.8° (2𝜃) corresponds to the (200) crystal planes of cellulose. The crystalline portion of the total contribution at 22.8° was calculated by the Segal method (Segal et al., 1959) and involved subtracting out the amorphous contribution at 18° (2𝜃). The latter was the peak position in the diffractogram of the amorphous sample. Calculation of the CI followed the equation CI (%) = I_200_-I_am_/I_200_ × 100, where I_200_ is the maximum intensity of the diffraction from the (200) plane at 𝜃=22.8° and I_am_ is the intensity of the amorphous background scatter measured at 2𝜃=18°.

### Determination of Flax Resistance to Fungal Pathogens

Firstly, flax seeds from transgenic plants and the control obtained in the field trial in each vegetation period were immersed in 96% ethanol for 1 min and then washed three times with sterile water and germinated on MS medium. Next the 7-day-old seedlings were transferred onto the medium (combined MS and PDA) with *Fusarium oxysporum* or *F. culmorum* (the fungi were cultured for 7 days at 18°C on potato-dextrose-agar (PDA) medium. At 10 days after transfer the numbers of infected flax seedlings (roots and hypocotyls) were counted and expressed as a percentage of the total plants used for the experiment (Starzycki, 1998).

### Preparation of Material for Microscopic Analyses

Ten stems (from 10 independent plants for each analyzed flax type) with complete growth and shaped capsules were collected. The material was cut into even sections (7 cm) and fixed in FAA mixture (37% formaldehyde 10 ml/acetic acid 5ml/70% ethanol 85 ml) used in anatomical studies of plants (Ruzin, 1999). Anatomical analysis of transverse sections was performed in the lower parts of sections (in the median portion) spreading from the cotyledonary node to the beginning of the apical branching zone of the main stem. Transverse sections were cut manually with a razor and stained with a mixture of Safranin 0 and Alcian blue (Gerlach, 1972). This method helps differentiate vascular tissues, phloem and xylem.

### Microscopic Analysis

Anatomical analyses of stems and seeds were conducted using: a Nikon ECLIPSE 80i with fluorescence and a Nomarski Interference Contrast microscope equipped with IKEGAMI DIGITAL type REV. B, model ICD-808P camera; a Nikon ECLIPSE TE2000-S inverted microscope and DIGITAL SIGHT DS camera; a Nikon SMZ 745T microscope with Moticam 5 MP camera; and a scanning electron microscope.

### Statistical Analysis

All of the experiments were independently performed in at least three technical repeats. The obtained results are presented as the averages of independent replicates ± SDs. Statistical analyses were performed using the STATISTICA v. 12 package (StatSoft Inc. 2014). The significance of the differences between means was determined using Student’s *t*-test. For each experiment *p*-values are given separately (*P < 0.05, **P < 0.01). For microscope analysis the normality of the data distribution was checked using the Shapiro–Wilk test. Spearman correlation was used for variables when the requirement of normality was not met.

## Results

### Characteristics of Transgenic Plants through Generations

Recently CHS down-regulated flax was obtained and characterized (Zuk et al., 2012). For transformation the construct containing CHS cDNA from *Petunia hybrida* in sense orientation under the control of the CaMV 35S promoter and OCS terminator was used. The seeds from transgenic plants showed a decrease in endogenous CHS gene expression which was accompanied by an increase in non-hydrolysable and hydrolysable tannin biosynthesis. The CHS –underexpressed plant indicated high antioxidant potential and subsequently higher resistance against *Fusarium* infection. An increase in polyunsaturated fatty acid accumulation was also detected. In this work transgenic plants of the 3rd, 5th, and 6th generation from three vegetation periods (2010, 2012, 2013) cultivated in the field were analyzed and compared to control plants, and data are presented in **Table 1**.

**Table 1:** Biochemical composition of seeds, antioxidant properties, and pathogen resistance of W86 plants across three generations of field cultivation.

	3th generation		5th generation		6th generation	
	Control	W86	Control	W86	Control	W86
CHS gene expression	100%	38% ± 2.5**	100%	41% ± 5.2**	100%	36% ± 3.4**
Anthocyanins [μg/gDW]	38.5 ± 0.8	68.5 ± 1.2**	40.3 ± 0.7	71.8 ± 1.6**	37.7 ± 0.6	70.9 ± 1.0*
Flavones [μg/gDW]	0.89 ± 0.06	0.99 ± 0.12	0.82 ± 0.08	0.89 ± 0.09*	1.02 ± 0.06	1.04 ± 0.06
Secoisolariciresinol diglucoside [mg/g DW]	182.3 ± 5.7	184.6 ± 4.3*	168.5 ± 5.1	199.1 ± 5.7*	179.9 ± 3.9	192.7 ± 5.6*
Proanthocyanins [mg/gDW]	0.3 ± 0.02	0.48 ± 0.01**	0.29 ± 0.02	0.53 ± 0.02**	0.31 ± 0.01	0.51 ± 0.02**
Hydrolyzable tannins [mg /gDW]	0.58 ± 0.07	2.7 ± 0.12**	0.59 ± 0.10	2.96 ± 0.18**	0.58 ± 0.21	2.76 ± 0.14**
Linoleic acid (18:2) [μg/gDW]	147 ± 3.34	110 ± 3.94*	136 ± 4.07	103 ± 5.7*	157 ± 5.54	92 ± 2.66**
αLinolenic acid (18:3) [μg/gDW]	4.43 ± 0.64	140 ± 6.54**	5.57 ± 0.7	132 ± 3.54**	6.06 ± 0.47	151 ± 5.11**
IC-50 [mg/ml]	0.27 ± 0.04	0.16 ± 0.04*	0.25 ± 0.05	0.12 ± 0.03*	0.27 ± 0.04	0.14 ± 0.02*
*Fusarium* infection	45%	23%**	34%	16%**	36%	21%*

Results are mean values ± SD (*n* = 6). Significance of differences between the means was determined using Student’s *t*-test (*P < 0.05, **P < 0.01).

Through three generations the total CHS gene expression level in transgenic plants varied only slightly (between 36 and 41% of the level of control plants) depending on the cultivation season.

The level of tannin components catechin gallate, epicatechin gallate, and proanthocyanidin B2 substantially increased (by 40%, 2.8-fold, and 34%, respectively), and levels of flavones and benzoic compounds underwent only slight changes (by about 11%–13%) compared to the control, which indicated the biosynthetic stability of the 6th generation transformants. Also a stable property of modified plants is the dark brown color of seeds originating from hydrolysable tannins accumulated in the testa. The higher level of tannins (hydrolysable tannins and proanthocyanins) compared to unmodified plants leads to an increase of transgenic plants’ antioxidant status. The increased antioxidant potential correlates with higher resistance of transgenic plants against *Fusarium* infection.

A very important feature of CHS-reduced plants, stable across the generations, is much higher stability of oil fatty acids than in controls. Consequently, transgenic plants produced more (20–45%) polyunsaturated fatty acids, and mainly α-linolenic acid resulted in a ratio of ω-6 to ω-3 fatty acids (1–2:1) in the oil, beneficial for the human diet.

The overall conclusion that can be drawn from biochemical analysis of the CHS-down-regulated plants is that their biochemical characteristics are stable across several generations. Transgenic plants of the 6th generation harvested at the end of the flowering stage (92nd day after sowing) were used throughout this work. At this stage the process of cell wall synthesis was completed or nearly completed and the plant stalk and fiber were fully developed.

### CHS Suppression Affects Cell Wall Constituent Biosynthesis

The major structural components (structural polymers) of the plant cell wall are cellulose, hemicelluloses, lignin and pectin. The amounts of these components determined in stalk from CHS-reduced transgenic flax (line W86) and a control plant (Linola) are presented in **Table 2**. The measurements of component compounds were accompanied by respective gene expression analysis.

**Table 2:** Content of major cell wall components determined in stalk from CHS-reduced transgenic flax (W86) and a control plant (Linola).

	Cellulose [mg/gDW]	Pectin [mg/gDW]	Hemicellulose [mg/gDW]	Lignin [mg/gDW]
Control	578.87 ± 48.8	35.8 ± 0.02	25.45 ± 0.02	21.3 ± 0.04
W86	654.78 ± 25.32*	35.2 ± 0.01	28.89 ± 0.05*	15.6 ± 0.013**

Results are mean values ± SD (*n* = 6). Significance of differences between the means was determined using Student’s *t*-test (*P < 0.05, **P < 0.01).

**Lignin** is a large complex of polymerized alcohol derivatives of phenolics such as coniferyl, sinapyl and p-coumaryl (Amthor, 2003). The data on lignin content (**Table 2**), its precursors (coumaric acid, ferulic acid, caffeic acid, coniferyl aldehyde) and other identified compounds from the same pathway (**Table 3**) as well as expression level (in comparison to control plants) of selected genes involved in biosynthesis of these compounds were presented.

**Table 3:** Lignin precursors and other identified compounds from phenylpropanoid pathway.

Compounds	Stalk control (μg/g DW)	Stalk W86 (μg/g DW)
Coumaric acid	7.56 ± 7.8	9.18 ± 4.39*
Ferulic acid	34.85 ± 4.7	87.5 ± 0.8**
Caffeic acid	6.37 ± 0.82	8.50 ± 1.09*
Syringic aldehyde	29.04 ± 1.34	28.94 ± 1.08
Syringic acid	12.75 ± 0.99	13.22 ± 1.4
Coniferyl aldehyde	86.63 ± 5.76	103.17 ± 12.89**
3,4-dihydroxybenzoic acid	47.86 ± 2.15	45.87 ± 2.1*
Vanillic acid	34.52 ± 0.1	39.27 ± 2.2**
Vanillin	161.77 ± 0.1	159.43 ± 4.7*
Vitexin	10.78 ± 0.46	11.99 ± 1.6*
Isoorientin	18.77 ± 4.77	20.87 ± 2.79*
Orientin	12.55 ± 4.94	13.26 ± 4.51
Proanthocyanidin B2	1.56 ± 0.31	2.09 ± 0.38**
Catechin hydrate	1.36 ± 0.06	1.51 ± 0.08**
Catechin gallate	1.13 ± 0.32	1.58 ± 0.18**
Epicatechin gallate	1.12 ± 0.09	3.16 ± 0.28**

Results are mean values ± SD (*n* = 6). Significance of differences between the means was determined using Student’s *t*-test (*P < 0.05, *P < 0.01).

The reduction (by about 48%) in CHS gene expression in mature flax plants was accompanied by considerably (almost twofold) higher expression of the PAL gene. The changes in phenylpropanoid compound contents and expression of respective genes was also noticed.

**FIGURE 1:**
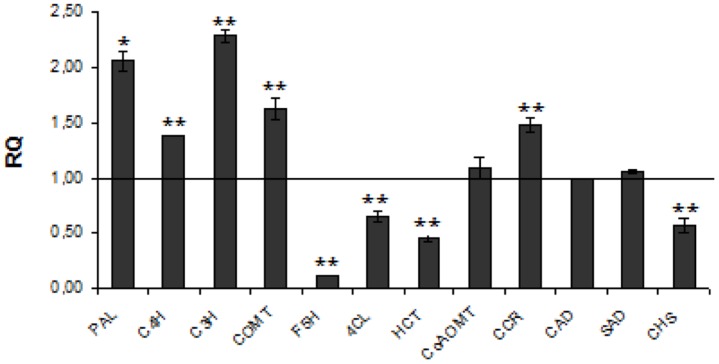
**Expression levels of phenylpropanoid pathway genes.** The mRNA level of phenylpropanoid pathway genes PAL, C4H, C3H, COMT, F5H, 4CL, HCT, CCoAOMT, CCR, CAD, SAD, CHS W86 flax in comparison with control flax obtained from the real-time RT-PCR analysis. The changes in transcript levels were presented as the RQ to the reference gene -actin. The transcript levels were normalized to those of the control plant (*C* = 1; not presented in the figure). Data represent the mean ± SDs from three independent experiments. The significance of the differences between the means was determined using Student’s *t*-test (*P < 0.05, **P < 0.01).

The detected increase in coumaric acid (by about 20%), caffeic acid (by about 33%) and ferulic acid (by about 2.5-fold) levels in transgenic plants might result from higher expression of cinnamic acid hydroxylase (C4H), p-coumarate-3-hydroxylase (C3H), and caffeic acid *O*-methyltransferase (COMT). At one time reduced expression of F5H and 4-hydroxycinnamoyl-CoA lyase (4CL) genes caused a slight decrease of the syringic aldehyde level. The increase in coniferyl aldehyde (by about 19%) corresponds to activation of CCoAMT and CCR genes. Although all these changes strongly suggest the increase in unit level for lignin biosynthesis, the lignin content was notably (by about 26%) reduced. The only genes from the lignin biosynthesis pathway that are significantly reduced in transgenic plants are 4CL, F5H, and *p*-hydroxycinnamoyl-coenzyme A: quinate shikimate p-hydroxycinnamoyltransferase (HCT). Thus F5H and HCT genes are somehow co-regulated with the CHS gene in lignin but not in flavonoid biosynthesis. This might suggest that biosynthesis of both groups, lignin and flavonoid compounds, in flax plants is regulated independently. The expression of genes involved in lignin metabolism is presented in **Figure 1**.

**FIGURE 2:**
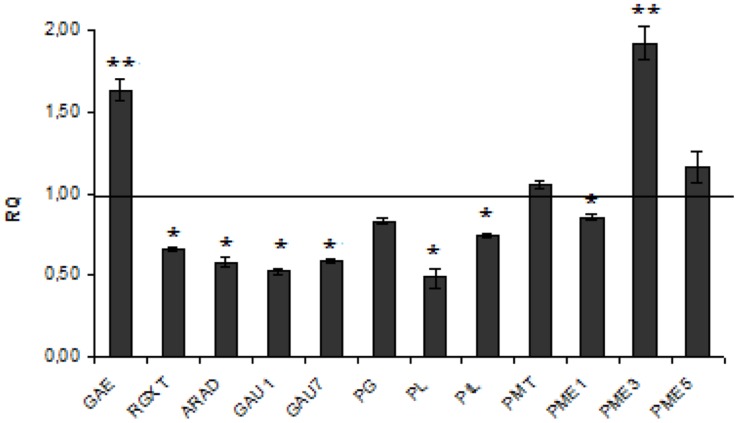
**Expression levels of pectin metabolism genes.** The mRNA level of pectin synthesis and degradation genes :GAE, RGXT, ARAD, GAU1,GAU7, PG, PL, PtL, PMT, PME1, PME3, PME5. W86 flax in comparison with control flax obtained from the real-time RT-PCR analysis. The changes in transcript levels were presented as the RQ to the reference gene -actin. The transcript levels were normalized to those of the control plant (*C* = 1; not presented in the figure). Data represent the mean ± standard deviations from three independent experiments. The significance of the differences between the means was determined using Student’s *t*-test (*P < 0.05, **P < 0.01).

**Pectin** is a saccharide polymer with galacturonic acid (70% of total) as a unit. Pectin plays a significant role in plant physiology and plant pathogen defense (Ma et al., 2013; Wojtasik et al., 2013, 2016). Of genes involved in pectin metabolism seven were down- and two up-regulated (see **Figure 2**). However, the level of polymer was unchanged in transgenic flax. This inconsistency might derive from the fact that the reduction (by about 33–48%) in expression of genes involved in synthesis is compensated by a decrease at a similar level (by about 26–52%) of those involved in pectin degradation (**Figure 2**). Two genes showed a substantial increase in expression: UDP-D-glucuronate 4-epimerase (by about 62%) involved in pectin synthesis and pectin methyltransferase 3 (by about twofold) involved in degradation. It thus suggests that the conversion of D-glucuronate to D-galacturonate is at a normal level but subsequent synthesis of homogalacturonan, rhamnogalacturonan I, and the substituted galacturonan rhamnogalacturonan II (RG-II) is perhaps disrupted. Higher pectin degradation promoted by the up-regulated PME3 gene perhaps compensates for its higher synthesis induced by UDP-D-glucuronate 4-epimerase.

**FIGURE 3:**
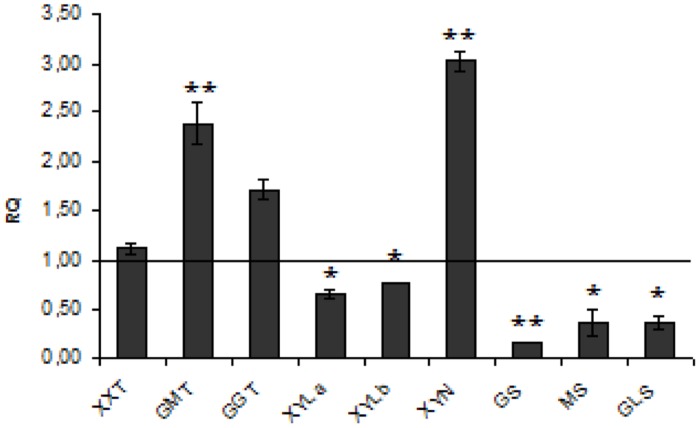
**Expression levels of hemicellulose metabolism genes.** The mRNA level of hemicellulose synthesis and degradation genes (XXT, GMT, GGT, XYLa, XYLb, XYN, GS, MS, GLS). W86 flax in comparison with control flax obtained from the real-time RT-PCR analysis. The changes in transcript levels were presented as the RQ to the reference gene -actin. The transcript levels were normalized to those of the control plant (*C* = 1; not presented in the figure). Data represent the mean ± SDs from three independent experiments. The significance of the differences between the means was determined using Student’s *t*-test (*P < 0.05, **P < 0.01).

**Hemicellulose** is a polymer composed of mixed xyloglucans, glucomannans, and glucuronoarabinoxylans. The process of hemicellulose biosynthesis is regulated by many enzymes from different branches of metabolism such as Golgi-localized glycosyltransferases (GTs), which facilitate the formation of the specific linkage between the monomers and thus synthesize the polymer, members of the cellulose synthase-like family A (CSLA) and mannan:galactosyltransferases, xylosyltransferases, and mannosyl transferases (Pauly et al., 2013). The full biosynthesis pathway of this polymer in flax is unknown. We measured the level of expression of three genes coding for the enzymes α-(1 → 6)-xylosyltransferase (XXT) which attaches D-xylose to the main chain of xyloglucans, glucomannan galactosyltransferase (GGT), and glucomannan β-mannosyltransferase (GMT), all of them involved in compound synthesis. In CHS-reduced plants a significant increase of GMT (by about 2.5-fold) and GGT (by about 65%) expression was detected. Concomitantly, decreased levels of expression of genes involved in hemicellulose degradation such as XYLa and XYLb, both catalyzing hydrolysis of terminal, non-reducing alpha-D-xylose or alpha-L-arabinofuranoside residues, were measured. The expression of other genes encoding GS, endo-β-mannosidase (MS), and β-glycosidase (GLS) was also significantly (by about 83, 64, and 65%, respectively) reduced. Of genes involved in hemicellulose catabolism, only the level of endo-β1,4-xylanase (XYN), which catalyzes the hydrolysis of (1 → 4)-beta-D-xylosidic linkages in xylans, was significantly (threefold) up-regulated. The expression of genes involved in hemicellulose metabolism is presented in **Figure 3**. The net effect of all these changes in gene expression was a 13.5% increase of hemicellulose content in CHS-down-regulated flax.

**Cellulose** is a linear polysaccharide consisting of β-1,4-linked D-glucose units, the core of the plant cell wall, serving as a scaffold for the other cell wall components (Wojtasik et al., 2016). CHS-reduced flax showed an increase in the cellulose content (by about 13%) compared to control plants.

**FIGURE 4:**
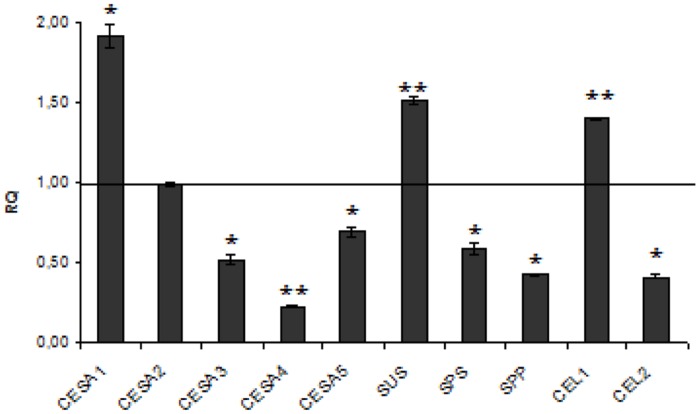
**Expression levels of cellulose metabolism genes.** The mRNA level of cellulose synthesis and degradation genes (CESA1, CESA2, CESA3, CESA4, CESA5, SUS, SPS, SPP, CEL1, CEL 2) W86 flax in comparison with control flax obtained from the real-time RT-PCR analysis. The changes in transcript levels were presented as the RQ to the reference gene -actin. The transcript levels were normalized to those of the control plant (*C* = 1; not presented in the figure). Data represent the mean ± standard deviations from three independent experiments. The significance of the differences between the means was determined using Student’s *t*-test (*P < 0.05, **P < 0.01).

The analysis of cellulose biosynthetic genes revealed that one of five CES genes (CES1) was activated and the others were not changed (CES2) or reduced (CES3, CES4, CES5). Also the cellulases genes were slightly induced (CEL1, by about 40%) or reduced (CEL2, by 2.5-fold). The genes coding for the cellulose precursor unit were also only slightly increased (SUSY, by about 51%) or reduced (SPS, SPP, by about 40 and 58%, respectively) (**Figure 4**). Thus the increase in cellulose content in W86 plants might have resulted from the prevalence of synthesis over compound degradation.

### CHS Down-Regulation Leads to Changes in Expression of Cell Wall Regulatory Genes

Suitable candidates for sensing function and signal transduction are transmembrane proteins. In transcriptional network, the secondary wall NACs, including SND, NST, and VND, are master switches turning on a subset of transcription factors, which in turn activate the secondary wall biosynthetic pathways (Mitsuda et al., 2007; Zhong et al., 2008). Some of wall associated kinases, such as WAK proteins, interact directly and strongly with pectin and structural protein in the cell wall. This observation corroborate its role in cell wall-sensing. (Park et al., 2001; Kohorn et al., 2009). FEI function in the regulation of cellulose biosynthesis and THE1 is involved in sensing cellulose deficiency and adapts cell wall development upon changes or irregularities in the cell wall structure (Ringli, 2010).

**FIGURE 5:**
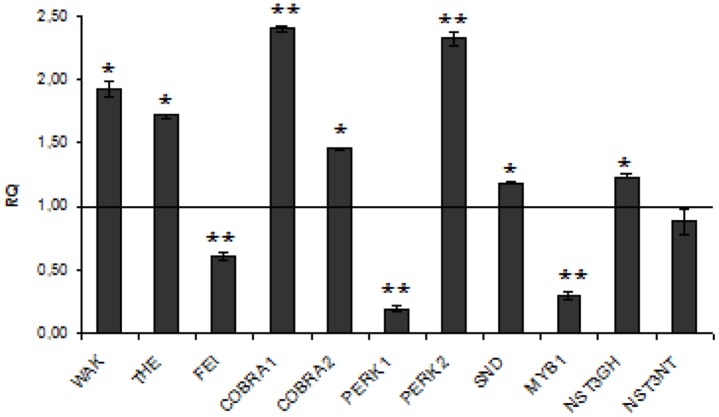
**Expression levels of cell wall sensing genes.** The mRNA level of cell wall sensing genes (WAK, THE, FEI, COBRA1, COBRA2, PRK1, PRK1, SND, NST3GH, NST3NT, MYB). W86 flax in comparison with control flax obtained from the real-time RT-PCR analysis. The changes in transcript levels were presented as the RQ to the reference gene -actin. The transcript levels were normalized to those of the control plant (*C* = 1; not presented in the figure). Data represent the mean ± standard deviations from three independent experiments. The significance of the differences between the means was determined using Student’s *t-*test (*P < 0.05, **P < 0.01). RQ – relative quantity.

The expression analysis of kinase genes (**Figure 5**) in W86 flax revealed that WAK, THE, and PERK2 are reasonably activated while FEI and PERK1 are strongly inhibited. The COBRA1 gene, which functions in relaying the positional information on the microtubules cytoskeleton to the cellulose synthase complexes and therefore is involved in establishing a continuum between the cytoskeleton and the cell wall, was significantly activated in transgenic flax.

Of NAC domain transcription factors SND and NST3GH were slightly induced while MYB1 was significantly reduced in transgenic flax. Since MYB is an important regulator of secondary wall biosynthesis in *Arabidopsis* (Zhong et al., 2007, 2008) and is able to bind to the promoters of lignin biosynthetic genes (Patzlaff et al., 2003; Goicoechea et al., 2005) we propose that its reduction in CHS-suppressed flax plants might be a direct reason for lignin deficiency therein.

### Wall Crystallinity Analysis

Cellulose microfibrils are the predominant constituent of the cell wall. Since all other polymers of the cell wall directly interact with cellulose, its structure thus reflects the arrangement of the entire cell wall (Keegstra, 2010; Scheller and Ulvskov, 2010). Crystallinity has an important effect on a wide range of cellulose properties. Several methods have been developed to analyze the arrangement of cellulose polymers, and X-ray scattering and Fourier transform infrared (FT-IR) spectroscopy are frequently used techniques. Both methods are suitable for cellulose crystallinity measurement. Decreasing crystallinity results in tensile strength, dimensional stability, and density decrease, while chemical reactivity and swelling increase (Agarwal et al., 1999, 2010).

**FIGURE 6:**
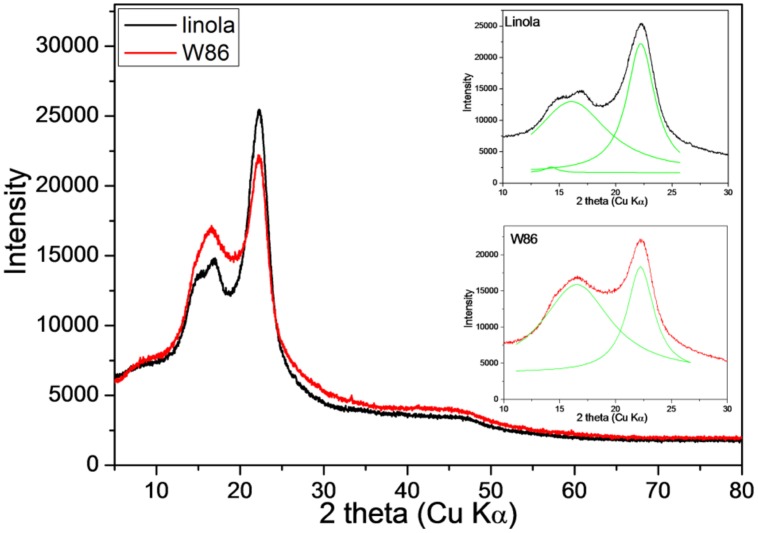
**X-ray diffraction diagram of engineered (red) and control plant (black) stalk.** Deconvolution of the 2 theta (CuKα) contour in the range of 10–30 into Lorentzian components (green) is inserted.

The typical cellulose diffraction pattern is dominated by equatorial reflections corresponding to (1–10), (110), and (200) planes according to the unit cell (Sugiyama et al., 1991). The 2𝜃 value of the peak corresponding to plane (200) for both transgenic and control cellulose is the same, but a significant decline in the peak heights was observed. The peak height was lower in the transgenic sample. It is proposed that the simple peak height method (Segal et al., 1959) is suitable for measurement of crystallinity values (Keshk, 2015). Using the respective equation it was calculated that the index crystallinity for the control is 50% while for the transgenic cellulose it is twofold lower (26.5%). Thus the result clearly suggests that CHS suppression resulted in more amorphous cellulose in flax (**Figure 6**).

However, in samples where hemicellulose, lignin, and pectin are present, their contributions cause extra scattering and lead to inaccurate measurement of the cellulose crystallinity by X-ray (Andersson et al., 2003).

**FIGURE 7:**
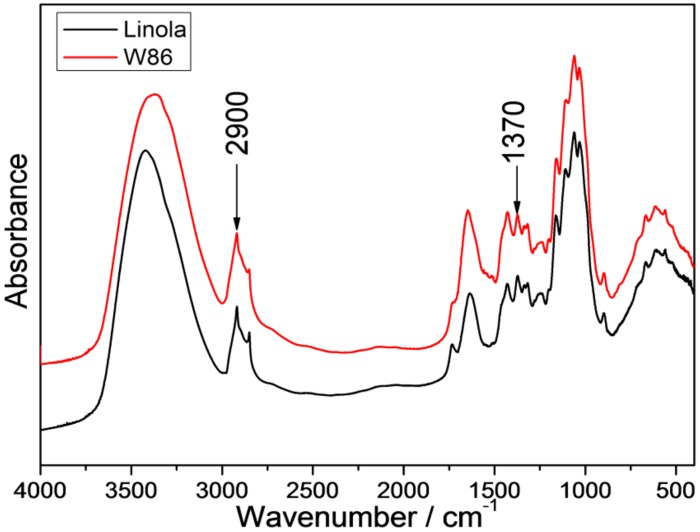
**IR spectra of stalk from engineered (red) and control flax (black) in the range of 4000 to 500 cm^-1^**.

Infrared spectra of cellulose were shown to be associated with a decline in crystallinity and/or decrease in the degree of polymerization of cellulose (Schwanninger et al., 2004). Therefore FT-IR or FT-Raman is commonly used as an alternative method for crystallinity measurements. For example, the weak (CH_2_) bending modes at 1462 and 1481 cm^-1^ in conjunction with spectral deconvolution have been used for cellulose I crystallinity quantization (Schenzel et al., 2005). However, considering the low intensities of these bands (**Figure 7**) and that the process of deconvolution is not free of band fitting problems, a better approach was sought. The spectra of the set of samples with different amorphous contributions were obtained and the peak height ratios for various bands were calculated. The band height ratios, were then plotted against the calibration set crystallinity, and it was found that for several peak ratios, where the intensity of the 2900 cm^-1^ band was used, the correlation coefficients were satisfied (Agarwal et al., 2010). Thus the band height ratios were determined for 1370 and 2900 cm^-1^ bands, and they were 1.213 for control and 1.081 for W86 transgenic plants (**Figure 7**).

The decline in lignin content might be the reason for lower crystallinity of the transgenic sample. This, however, needs to be verified experimentally.

### Cell Wall and Stem Anatomy

#### Stem Microscopy

**FIGURE 8:**
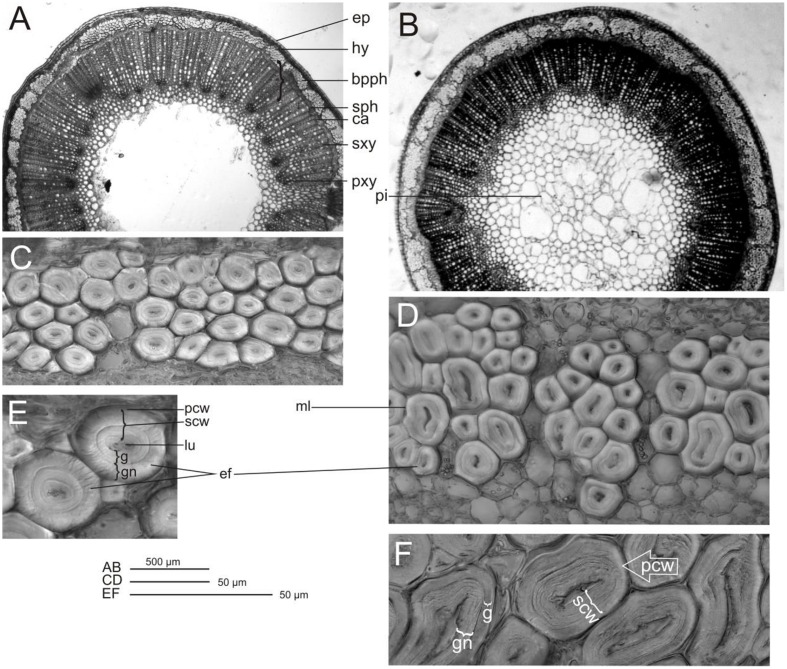
**Control and modified flax stem and fiber microscopy. Cross-section of flax stem control (A)**, modified form W86 **(B)**; phloem fibers: control **(C)**, modified form W86 **(D)**, and elementary phloem fibers: control **(E)**, modified form W86 **(F)**; epidermis (ep), hypnodermis (hy), bundle of primary phloem fibres (bpph), secondary phloem (sph), cambium (ca), secondary xylem (sxy), primary xylem (pxy), pith (pi), elementary fibres (ef), lumen (lu), secondary cell wall (scw), primary cell wall (pcw), middle lamellae (ml) gn – newly deposited gelatinous layer of secondary cell wall, g – mature gelatinous layer of secondary cell wall.

Transverse sections of stems from control flax plant vary from circular and smooth to slightly waveform, particularly near the apical branching zone. The wavy surface of the shoot perimeter is additionally strengthened by triangular cells in the rind, which have a cuticle point at the top. The mean cross-sectional area for this variety is 3.85 mm^2^, with a diameter of 1.25 mm. CHS-reduced plants show a regular round shape and the stem perimeter is smooth. The mean cross-sectional area is 7.55 mm^2^, with a diameter of 3.1 mm. Thus the cross-sectional area of the transgenic stem is almost twice as high as that of the control (**Table 4**). The cross-section of both plant types (control and transgenic) showed a relatively large pith, broad primary and secondary xylem rings and primary and secondary phloem. The primary phloem with numerous big bast fiber bundles is clearly visible. Between the xylem and phloem there is a narrow ring of cambium. In the cross section perimeter there is epidermis and hypodermis, and a layer of cortex cells. Stem epidermal cells show some differences in wall thickness. The epidermis of control plants consists of cells with a conspicuously thickened outer wall, but for W86 flax type the external and internal surfaces are equally thick (**Figures 8A,B**).

**Table 4:** Anatomical traits of Linola (control) and W86 stems.

	Control	W86	W86/control
Stem area [mμm^2^]	3.86 ± 0.31	7.55 ± 0.30**	1.96
In stem cross-section:
Cortex with epidermis area [mm^2^]	0.42 ± 0.07	0.56 ± 0.08	1.35
Total phloem area [mm^2^]	0.59 ± 0.05	1.35 ± 0.18**	2.31
Fiber bundle area [mm^2^]	0.37 ± 0.03	0.86 ± 0.10**	2.33
Secondary phloem and cambium area [mm^2^]	0.21 ± 0.03	0.49 ± 0.10**	2.28
Xylem area [mm^2^]	1.82 ± 0.24	3.57 ± 0.87*	1.96
Pith area [mμm^2^]	1.03 ± 0.12	2.07 ± 0.91*	2.00
Total no. of fiber cells	1255 ± 243	2917 ± 501**	2.32
Fiber area [μm^2^]	270.41 ± 116.52	666.22 ± 289.08**	2.5
Fiber wall area [μm^2^]	255.23 ± 112.13	638.59 ± 278.06**	2.5
Lumen area [μm^2^]	15.18 ± 11.05	27.63 ± 21.70**	1.8

Results are mean values ± SD (*n* = 10). Normality of the data distribution was checked using the Shapiro–Wilk test. Spearman correlation was used for variables when the requirement of normality was not met. (*P < 0.05, **P < 0.01).

The area of all tissues of modified flax was 1.96 to 2.33 times larger than for control plants (the only exceptions are the cortex and epidermis, where this increase is only 1.35-fold) (**Table 4**). The highest increases (by about 2.3-fold) were observed for total phloem, fiber area and secondary phloem with cambium area. The phloem and xylem together have over 60% of cross section area in both flax types. The ratio of phloem to xylem was 0.37 and 0.32 for modified flax and control, respectively. This confirms that the W86 flax has a larger share of phloem in the stem and, at the same time, less xylem. The increase of phloem is a result of a larger single fiber area and higher number of fiber cells in the stem (**Figures 8C,D**).

Fiber microscopy In both flax types primary fibers occur in tightly packed groups (phloem fiber bundle). Bundle fibers separate the medullar rays, which are of varying width. Analyses have shown that the W86 type compared to the control has 1.4 times bigger cells and 2.8 times bigger lumen and thus a bigger wall surface area (**Table 4**). The cross section of fibers shows the lamellar structure of walls (alternating cellulose and non-cellulose layers). A difference is detected in the inner part of the second wall, which shows a more loosely layered structure (gn-layer) in comparison to the outer part, which has a homogeneous uniform structure (g-layer). Control fiber is characterized by similarly broad g layers with dense structure (mature layer cell wall with cellulose microfibrils and poor in galactan) and gn with looser structure (newly deposited layer cell wall). The secondary walls of the W86 fiber have a relatively thin g layer and a broad gn layer with clear broad layers of loosely packed cellulose microfibrils separated by thin dark stripes perhaps of galactans (**Figures 8E,F**).

## Discussion

Many biochemical pathways in plants show a tendency to be flexible and to be responsive to environmental changes. This is related to the fact that plants cannot avoid environmental stress. Also a flexible biochemical pathway allows optimization of the use of limiting resources. One of the best examples is the phenolics pathway (Li et al., 2010). Plant phenolics fulfill important functions, being involved in development and interaction of the plant with its environment. Their particular functions are varied and range from scent, pigment and poisons through signaling and structural molecules to antibacterial and antifungal features. The phenolics are grouped into different classes based on their structure, and the most abundant are simple phenolics which are a mixed group of simple phenylpropanoids and benzoic acid derivatives, flavonoids, lignin, and tannins. The shikimic pathway leads to the production of the major plant phenolic groups. Shikimic acid is formed from erythrose 4-phosphate (pentose phosphate pathway) and phosphoenolpyruvate (glycolysis). Next seven reactions result in the formation of chorismic acid, which is the start point for aromatic amino acid and gallic acid synthesis. *Trans*-cinnamic acid, which is the product of phenylalanine conversion catalyzed by PAL enzyme, is the branch point for *p*-coumaric acid (reaction catalyzed by cinnamate 4-hydroxylase) and benzoic acid derivatives. *p*-Coumaric acid might be converted to coumaroyl-CoA by 4CL and further by cinnamate 3-hydroxylase (C3H) and hydroxycinnamoyl-CoA shikimate/quinate hydroxycinnamoyl transferase (HCT) to lignin or by CHS to chalcones and then to proanthocyanidin and flavonoids.

The rapid responses of plants to environmental signals such as pathogen infection, wounding and UV light are all due to CHS gene expression induction and flavonoid production. The same role, however, is ascribed to other enzymes and their products. These include PAL induction and synthesis of benzoic acid derivatives, isochorismate synthase activation and salicylic acid biosynthesis, and accumulation of tannins and lignin. Although they share similar biological functions, they are structurally and biosynthetically quite distinct.

To determine more precisely the role of CHS in the phenolics pathway and the cell wall, and the structural consequences of its action, the gene expression profile, biochemical analysis and cell wall morphology parameters approach for transgenic plant investigation was used. In flax, CHS gene silencing (W86 plant) has been shown to lead to profound changes in phenylpropanoid metabolism. For example, lignin biosynthesis was inhibited, but non-hydrolysable tannin (proanthocyanidin) and hydrolysable tannin contents were increased. Since both tannin and lignin compounds issue from the phenylpropanoid pathway and use the same precursor (p-coumaryl CoA), co-regulation of their biosynthesis, i.e., rerouting the substrates from lignin biosynthesis to other routes of the phenolic biosynthesis pathway, is suggested.

The data from *Arabidopsis* support this view. For example, suppression of key lignin biosynthesis genes (HCT, C3H) in this plant results in lignin synthesis reduction, which leads to redirection of the metabolic flux into flavonoids through CHS activity. This, however, is not the case in eucalyptus plants (*Eucalyptus globulus*), where a positive correlation was found between HCT transcript level and lignin content, while no such correlation between CHS transcript content and lignin and flavonoid levels was detected (Shinya et al., 2014). This might suggest that biosynthesis of both groups, lignin and flavonoid compounds, in eucalyptus plants is regulated independently. Flax plants with suppression of key flavonoid biosynthesis gene (CHS) demonstrate decline in lignin content, with only small changes in flavonoids. In both developmental stages – seedlings (data not shown) and mature plants – lignin synthesis dominates over flavonoid synthesis. The ratio between the former and latter being 6–8 in young plants and 25–30 in mature plants, which indicates a typical gene dosage effect (the greater the number of genes, the greater their biological effect). It is thus possible that the level of CHS activity in the non-transgenic plants is in great excess and in transgenic flax at a sufficient level required for normal flavonoid biosynthesis but insufficient for activity leading to other biosynthetic routes of phenylpropanoids, including lignin. Indeed, concomitant 4CL, HCT, and F5H gene suppression in CHS-silenced flax plants has been detected. The molecular background for this co-regulatory effect is, however, as yet unknown.

Several reports suggest that the changes in cell wall constituents induce a compensatory mechanism (for review see Ringli, 2010). For example, in *Arabidopsis thaliana* mutation in cellulose synthase CesA genes leads to a reduction in cellulose content and induces modifications in lignin and pectin deposition. Thus, cellulose deficiency was recognized as a cell wall-sensing process which induces an appropriate response (Bosca et al., 2006; Hernandez-Blanco et al., 2007). It might thus be suggested that cell wall sensing and induction of a compensatory mechanism are perhaps also the reasons for the increase in cellulose and hemicellulose in lignin-deficient W86 flax. In this plant, slight induction of the CES1 gene but a significant reduction of the cellulase gene (CEL2) has been detected. Since the genes controlling the synthesis of the cellulose precursor unit (SUSY, SPS, SPP) were not substantially affected, the increase in cellulose content in W86 plants might have resulted from the prevalence of polymer synthesis over degradation through reduction of the cellulase gene. Perhaps the most suitable candidates for sensing function and signal transduction are transmembrane proteins (Ringli, 2010). Cell wall and extracellular matrix associated kinases (WAK, THE, FEI, PERK, COBRA) and NAC domain transcription factors (NST, SND, MYB) beside involvement in cell expansion, are also induced upon pathogen attack or as a stress response (Sivaguru et al., 2003). It is interesting that WAK proteins interact strongly with pectin (Kohorn et al., 2009) and also with the Gly-rich protein GRP3, a structural protein in the cell wall (Park et al., 2001). This indicates direct interaction of WAK with the cell wall, and makes it a reasonable candidate for a cell wall-sensing function. The expression analysis of kinase genes in W86 flax reveals that most of the identified genes are activated. Of other potential sensing proteins, only the COBRA gene, which functions in relaying the orientation of the cortical microtubules to the movement of cellulose-synthesizing rosette complexes, was significantly activated in transgenic flax. All this might suggest that a network of kinases and signal transducing proteins is involved in the regulation of cell wall biosynthesis. For example it was previously indicated, that THE1 is involved in sensing cellulose deficiency and adapts cell wall development upon changes or irregularities in the cell wall structure (Ringli, 2010). In this regulatory network, kinases such as PERK, THE, and COBRA transducer may be part of a group of proteins that act as sensors/receptors at the cell wall. These sensors/receptors, for example, may monitor changes to the cell wall during cell expansion in plant growth, or during plant exposure to abiotic/biotic stresses, and activate associated cellular responses (Roudier et al., 2005; Doblin et al., 2014). This suggestion is additionally supported by the study of *Arabidopsis* plants. Overexpression of all NST genes leads to ectopic deposition of secondary walls in cells that are normally parenchymatous, and silencing of their activity results in a reduction in secondary wall thickening (Zhong et al., 2007). In transgenic W86 plants, of NAC domain transcription factors, MYB was reduced and others (SND, NST) not changed. MYB transcription factors have also been shown to be important regulators of secondary wall biosynthesis in *Arabidopsis* (Zhong et al., 2007). MYB is a direct target of SND kinase, and is able to turn on the biosynthetic pathways of cellulose, xylan, and lignin. MYB genes, such as those from *Pinus taeda* and from *Eucalyptus grandis*, were able to bind to the AC elements present in the promoters of lignin biosynthetic genes and are proposed to regulate the biosynthesis of lignin (Patzlaff et al., 2003; Goicoechea et al., 2005). Thus down-regulation of MYB in flax might be a direct reason for lignin deficiency in CHS-suppressed plants.

In *Arabidopsis thaliana*, silencing of lignin biosynthetic genes (HCT, C3H) leads to redirection of the metabolic flux into flavonoids through CHS activity and results in a strong reduction of plant growth. Suppression of CHS in HCT-deficient plants restored normal plant growth and thus suggests that reduced size phenotype of plants is not due to the alteration of lignin synthesis but to flavonoid accumulation (Besseau et al., 2007). C3H-deficient plants, like HCT-deficient ones, are also dwarf and accumulate high levels of flavonoids. However, blocking flavonoid biosynthesis with a CHS null mutation had no effect on the growth phenotype (Li et al., 2010). In addition to C3H and HCT, silencing of other genes of the phenylpropanoid pathway also results in plant growth reduction but without flavonoid hyper-accumulation or in some cases with reduction of these metabolites. These are for example tobacco with PAL deficiency, alfalfa with suppression of C4H (cinnamic acid 4-hydroxylase) and *Arabidopsis* ref3 and irx4 mutants (Jones et al., 2001; Reddy et al., 2005; Schilmiller et al., 2009).

Lignin deficiency in CHS-reduced flax does not affect plant growth. However, several abnormalities in stem morphology have been detected. For example, a cross section of the stem revealed that the area of all tissue layers was enlarged at least twofold compared to the control. It is interesting that the number of fiber cells is also increased by more than twofold and thus resembles fibrous rather than linseed flax stem. Based on this feature, the W86 linseed plant appears as a real dual-purpose plant providing beside oilseed also fiber in yields characteristic for fibrous flax, which is beneficial from an industrial point of view. The reason for the normal W86 plant height and thick culms is as yet unknown. However, the increased cellulose content in this plant and its structural arrangement might at least partially account for this. Cellulose crystallinity is an important parameter of polymer properties (Li et al., 2014). The changes in this parameter affect physical (tensile strength, dimensional stability, density decrease) and chemical (reactivity, swelling) features.

X-ray and FT-IR are powerful techniques commonly used to describe the crystalline structure of materials. Their applications to cell wall investigation include the determination of the degree of crystallinity of cellulose, which governs the mechanical and chemical properties of the material. Both methods can provide a rough estimation of crystallinity through measuring the relative crystalline index, which is based on the proportions of crystalline and amorphous cell wall material. The X-ray patterns of control and transgenic samples clearly show that the crystallinity of transgen cellulose is significantly reduced. This finding was confirmed by FT-IR measurements. Thus CHS-suppressed flax showed a reduction in this parameter, suggesting that cellulose polymers are in a more amorphous state than in control plants. This is in agreement with the suggestion that X-ray measurement can be influenced by non-cellulosic polysaccharides (Park et al., 2010; Harris et al., 2012). Thus, lignin level reduction in CHS-reduced flax is perhaps a direct signal for a more disordered structure of cellulose and the entire cell wall in transgenic plants. This suggestion is fully in agreement with the data from the microscopy study.

In summary, in this study a further insight into the function of the CHS gene was provided by a detailed analysis of the flax gene. A CHS-silenced plant accumulates tannins, shows a decline in lignin and an increase in cellulose synthesis, and grows normally, although some abnormalities in stem morphology were detected. Since the flax stem is the source of fiber, its cell wall composition determines fiber quality and its potential. For example, a decrease of lignin in the plant stem improves fiber resilience, which is beneficial for textile production and additionally enhances the potential of stem residual (after fiber extraction) for methane production (Wróbel-Kwiatkowska et al., 2015).

**FIGURE 9:**
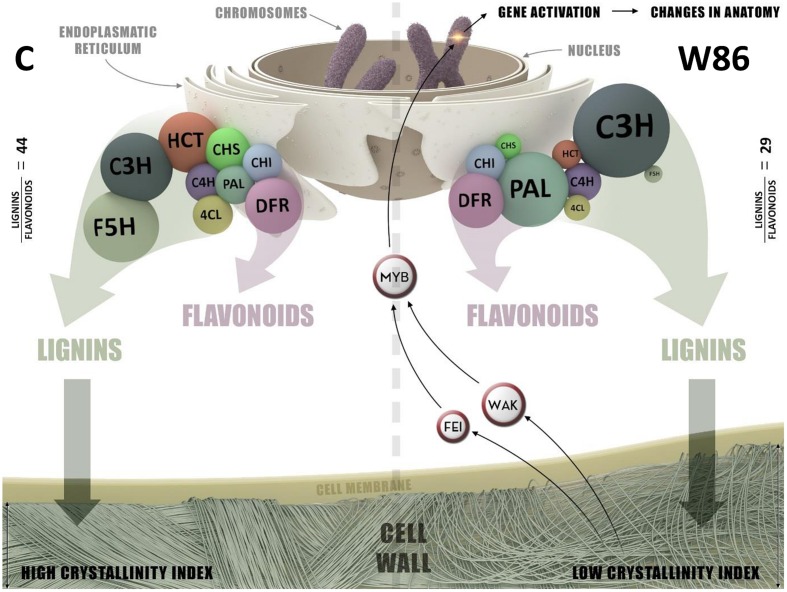
**The graphical model of enzymatic complex engaged in regulation of metabolic pathways of synthesis phenylpropanoids and lignin in control (C) and W86 flax plant**.

There is a lack of understanding about the steps between flavonoid/lignin blocking and *Arabidopsis* dwarf phenotype or flax stem abnormalities. We have assessed this in some cases by cell wall gene expression analysis and measurements of cell wall constituents. CHS gene silencing redirects flavonoid substrates to synthesis of tannins, and reduces lignin synthesis, which induces a compensatory mechanism of cellulose synthesis. Significantly reduced cellulose crystallinity is perhaps the direct cause of detected stem abnormalities. The signaling role of CHS is based on the participation of the enzyme in lignin synthesis. Pathways of flavonoid and lignin synthesis are carried out by multi-enzymatic complexes, or perhaps a single complex, maintaining cohesion due to the particular structure of their constituents and, more importantly, the existence of an “organizer” for the complexes. CHS may constitute such an organizer through its direct or indirect (e.g., by means of C4H or CHI ability to anchor the rough endoplasmic reticulum (ER), as was suggested many years ago based on CHS immunolocalization, e.g., in material collected from mustard plants (Hrazdina and Wagner, 1985; Jørgensen et al., 2005). The lipophilic character of chalcone, the product of CHS activity, makes a close relationship between CHS and biological membranes more probable. The graphical model of the hypothetical enzymatic complex engaged in regulation of metabolic pathways of synthesis of phenylpropanoids and lignin in flax plants is presented in **Figure 9**. A decreased amount of CHS may signal such a complex to break up or reduce its own activity within certain metabolic pathways. The reduced activity of this complex in lignin synthesis may stem from the polarization of its constituents: CHS is more closely associated with consecutive flavonoid enzymes, e.g., CHI or DFR, and less closely with HCT, which allows metabolites to flow more easily along the flavonoid pathway compared to the lignin pathway. Alternatively, two enzymatic complexes may coexist, competing over substrates that synthesize flavonoids and lignins. The varied function of these complexes (synthesis of flavonoids or lignins) would stem from their composition. Both complexes may contain the PAL enzymatic proteins common for both pathways, i.e., C4H and 4CL, and an izoform of CHS that determines further association with the enzymes of flavonoid (CHI, DFR, etc.) or lignin (C3H, HCT, F5H, etc.) synthesis and anchors the complexes in the ER.

## Conclusion

Chalcone synthase silencing induces a signal transduction cascade that leads to extensive modification of plant metabolism and thus cell wall structure. It is thus possible that the level of CHS activity in transgenic flax is at a sufficient level required for normal flavonoids biosynthesis but insufficient for the activity leading to other biosynthetic routes of phenylpropanoids, including lignin. Lignin level reduction in CHS-reduced flax is perhaps a direct signal for a more disordered structure of cellulose and the entire cell wall in transgenic plants. This suggestion is fully in agreement with the data from the morphological study. Significantly reduced cellulose crystallinity is perhaps the direct cause of detected stem abnormalities.

## Author Contributions

MZ, carried out the biochemical analysis of plants, cell wall polymers levels, and participated in writing of the manuscript; MD, performed gene expression analysis; DR, performed anatomical analysis; LD, carried out FT-IR and X-ray analysis of stem; JM, participates in interpretation of anatomical analysis and preparation of anatomical part of study; AK was responsible for flax field breading; JH, participates in interpretation of FT-IR and X-ray analysis and participate in preparation of spectroscopic part of study; JS, conceived of the study and participate in its design and coordination. All authors read and approved the final manuscript.

## Conflict of Interest Statement

The authors declare that the research was conducted in the absence of any commercial or financial relationships that could be construed as a potential conflict of interest. The reviewer JM and handling Editor declared their shared affiliation, and the handling Editor states that the process nevertheless met the standards of a fair and objective review.

## Footnotes

1 http://www.ncbi.nlm.nih.gov/blast/

## Abbreviations

4CL: 4-coumarate-CoA ligase
ARAD: arabinosyltransferase
C3H: p-coumarate 3-hydroxylase
C4H: cinnamate-4-hydroxylase
CAD: cinnamylalcohol dehydrogenase
CCoAOMT: Caffeoyl-CoA O-methyltransferase
CCR: cinnamoyl-CoA reductase
CEL 2: cellulase 2
CEL1: cellulase 1
CESA1: cellulose synthase 1
CESA2: cellulose synthase 2
CESA3: cellulose synthase 3
CESA4: cellulose synthase 4
CESA5: cellulose synthase5
CHI: chalcone isomerase
CHS: chalcone synthase
COBRA1: GPI-anchor protein1
COBRA2: GPI-anchor protein 2
COMT: Caffeate O-methyltransferase
DFR: dihydroflavonol reductase
F5H: ferulate 5-hydroxylase
FEI: LRR receptorlike serine/threonine-protein kinase
FT-IR: Fourier transform infrared spectroscopy
GAE: UDP-D-glucuronate-4-epimerase
GAU1: alpha-1,4-galacturonosyltransferase 1
GAU7: alpha-1,4-galacturonosyltransferase 7
GGT: galactomannan galactosyltransferase
GLS: b-glycosidase (cellobiase)
GMT: glucomannan 4-beta-mannosyltransferase 9-like
GS: -galactosidase
HCT: 4-coumaroyl-CoA:shikimate O-(hydroxycinnamoyl) transferase
MS: endo-b-mannosidase
MYB: MYB DNA binding domain transcription factor
NST3GH: Gossypiumhirsutum NAC trascription factor 3
NST3NT: Nicotian tabacum cultivar SR1 NAC secondary wall thickening promoting factor 3
PAL: phenylalanine/tyrosine ammonia-lyase
PG: polygalacturonase
PL: pectinlyase
PME1: pectinmethylesterases 1
PME3: pectinmethylesterases 3
PME5: pectinmethylesterases 5
PMT: pectinmethyltransferase
PRK1: prorichextensin-like receptor kinase 1
PRK1: pro-richextensin-like receptor kinase 2
PtL: pectatelyase
RGXT: rhamnogalacturonan II xylosyltransferase
RQ: relative quantification
SAD: synapyl alcohol dehydrogenase
SND: Domain transcription factor SND1
SPP: sucrose phosphate phosphatise
SPS: sucrosephosphate synthase
SUS: sucrose synthase
THE: receptor-like protein kinase THESEUS
WAK: wall-associated kinase
X-ray: X-Ray Diffraction.
XXT: xyloglucanxylosyltransferase
XYLa: 1,4-alpha-xylosidase
XYLb: 1,4-b-xylosidase
XYN: endo-1,4-b-xylanase.
